# Wie kann die personenzentrierte zahnmedizinische Versorgung nachhaltig gefördert werden?

**DOI:** 10.1007/s00103-021-03380-3

**Published:** 2021-06-25

**Authors:** Stefan Listl, Ingrid Schubert

**Affiliations:** 1 Lehrstuhl für Qualitätsförderung in der Zahnmedizin, Radboud University Medical Center, Radboud Institute for Health Sciences, Nijmegen, Niederlande; 2 Sektion für Translationale Gesundheitsökonomie, Poliklinik für Zahnerhaltungskunde, Universitätsklinikum Heidelberg, Heidelberg, Deutschland; 3 PMV forschungsgruppe an der Klinik und Poliklinik für Psychiatrie, Psychosomatik und Psychotherapie des Kindes- und Jugendalters, Medizinischen Fakultät und Uniklinik Köln, Universität zu Köln, Köln, Deutschland

Im ersten Heft zum Thema Mundgesundheit (Heft 7/21) haben wir den Bogen von der Epidemiologie über Versorgungsaspekte hin zur zahnmedizinischen Versorgungsforschung gespannt. In diesem Heft finden Sie Beiträge, die sich mit dem individuellen und gesellschaftlichen Wert der Mundgesundheit befassen. Thematisiert werden ausgewählte zahnmedizinische Erkrankungen (auch unter der Perspektive der Psychosomatik), Aspekte der mundgesundheitsbezogenen Lebensqualität und der partizipativen Entscheidungsfindung, Kosten der zahnmedizinischen Versorgung sowie Erwägungen zu ressourcenschonenden Versorgungsalternativen. In vielen Bereichen – das zieht sich wie ein roter Faden durch die Beiträge – konstatieren die Autorinnen und Autoren noch weiteren Forschungsbedarf und sehen die Notwendigkeit einer besseren Translation wissenschaftlicher Erkenntnisse in geeignete gesundheitspolitische Rahmenbedingungen und konkrete Versorgungsformen.

Zahn- und Kieferfehlstellungen gehören, wie *Sabine Ruf, Peter Proff und Jörg Lisson* in ihrem Beitrag darlegen, zu den häufigsten Mundgesundheitsbeeinträchtigungen beim Menschen. Die Autorengruppe erläutert die multifaktoriellen Ursachen und zeigt präventive und kurative Behandlungsmöglichkeiten auf. Das Evidenzniveau zum Effekt kieferorthopädischer Behandlungen auf die Mundgesundheit wird als vergleichsweise gering beschrieben. Auch liegen national wie international bislang nur relativ wenige Studien zur Qualität kieferorthopädischer Maßnahmen vor.

Hohe Prävalenzen von rund 14 % bis 17 % bei 10- bis 15-Jährigen ermittelten *Jan Kühnisch et al.* hinsichtlich der Molaren-Inzisiven-Hypomineralisation, einer Störung unbekannter Ätiologie bei der Bildung des Zahnschmelzes (sog. Kreidezähne). Für die Betroffenen ist dies mit funktionellen und ästhetischen Beeinträchtigungen verbunden. Die Autoren betonen die Notwendigkeit einer Ursachenforschung, um entsprechende Präventionskonzepte entwickeln zu können.

Parodontitis wird aufgrund ihres Ausmaßes als Volkskrankheit bezeichnet. *Bettina Dannewitz, Birte Holtfreter und Peter Eickholz* erläutern das Krankheitsbild und zeigen auf, dass eine Behandlung oftmals zu spät in Anspruch genommen wird mit der Folge einer schlechteren Prognose für den Erhalt der Zähne. Ihres Erachtens besteht in der Bevölkerung ein unzureichendes Wissen über die Erkrankung und ihre Risikofaktoren sowie über die Möglichkeiten der Behandlung. Auch sollte der Parodontologie ein höherer Stellenwert in der universitären Ausbildung zukommen und die postgraduale Ausdifferenzierung von Spezialisten oder Fachzahnärzten für Parodontologie vorangetrieben werden. Durch die am 01.07.2021 in Kraft tretende neue Behandlungsrichtlinie bestehen nach Ansicht der Autorengruppe verbesserte Rahmenbedingungen für die Behandlung der Parodontitis und anderer Parodontalerkrankungen.

Der Beitrag von *Lina Jansen et al*. befasst sich mit Lippen‑, Mundhöhlen- und Pharynxkarzinomen. Die Autorengruppe berichtet unter Rückgriff auf die Datenbank des Zentrums für Krebsregisterdaten (ZfKD) Inzidenz- und Mortalitätsdaten für Deutschland für die Jahre 1999–2016. Beobachtet wurde eine deutliche Tendenz zu höheren Inzidenz- und Mortalitätsraten bei Männern im Vergleich zu Frauen. Frauen wiesen eine höhere relative 5‑Jahres-Überlebensrate als Männer nach Mundhöhlen‑, Speicheldrüsen- und Oropharynxkarzinomen auf. Hierzu werden erklärende Hypothesen berichtet.

Zahnmedizinische Beschwerden werden häufig als primär somatisch erklärt. Dies greift, wie *Anne Wolowski, Hans-Joachim Schneider und Thomas Eger* in ihrem Beitrag zu zahnmedizinischen Beschwerdebildern mit psychosomatischem Hintergrund aufzeigen, oftmals zu kurz. Es geht hierbei um mehr als die Angst vor der zahnärztlichen Behandlung. Die Autorengruppe beschreibt einige zahnmedizinische Erkrankungen, bei denen die Berücksichtigung psychosozialer Aspekte den Erfolg der Behandlung verbessern kann. Diese Perspektive erfordert eine stärkere Sensibilisierung aufseiten der Zahnmedizin für psychosomatische Aspekte sowie in der Regel auch eine enge Zusammenarbeit mit anderen medizinischen Fachdisziplinen.

Mit Mundgesundheit aus der Perspektive von Patienten befassen sich zwei weitere Beiträge. *Danna R. Paulson et al*., eine Autorengruppe aus Minnesota, geben einen Überblick, wie Mundgesundheit aus Patientenperspektive erfasst und in die Entscheidungsfindung für klinische wie auch Public-Health-Interventionen einbezogen werden kann. Hierzu beschreiben sie, wie die vier Dimensionen der mundgesundheitsbezogenen Lebensqualität (Funktion, Ästhetik, Schmerz, psychosozialer Einfluss) mittels eines universellen Messwerkzeugs erfasst werden können. *Laura Krause, Stefanie Seeling und Anne Starker *konnten auf Basis einer bundesweit repräsentativen Stichprobe für die erwachsene Bevölkerung in Deutschland die selbstwahrgenommene Mundgesundheit einschließlich assoziierter Faktoren erheben. Fast 29 % der Befragten beschreiben ihre Mundgesundheit als mittelmäßig bis sehr schlecht. Bei Männern liegt dieser Anteil höher als bei Frauen und es zeigt sich insgesamt eine negative Korrelation zum Bildungsstand. Ein Zusammenhang zwischen schlechter selbstberichteter Mundgesundheit und geringer Mundgesundheitskompetenz wird auch in der Literatur beschrieben.

Damit ist das Stichwort für zwei Beiträge zur Mundgesundheitskompetenz gegeben. Eine Autorengruppe um *Kristin Spinler* befasst sich mit Mundgesundheitskompetenz von Menschen mit Migrationshintergrund. Sie konnten in ihrer Erhebung in 40 Zahnarztpraxen aufzeigen, dass der Migrationshintergrund auch unter Kontrolle für sozioökonomische und Bildungsfaktoren einen eigenständigen Indikator für eine relativ niedrige Mundgesundheitskompetenz und schlechtere Mundgesundheit darstellt. Nach Ansicht der Autorengruppe bedarf es hier zielgruppenspezifischer Ansprache. Im Rahmen ihres Projektes wurde eine Mundgesundheitspräventions-App in verschiedenen Sprachen eingesetzt.

Gesundheitsinformationen zu vermitteln und Gesundheitskompetenz zu verbessern, wird als gesamtgesellschaftliche Aufgabe gesehen, der sich auch die Zahnmedizin stellt. Hierzu bedarf es des Gesprächs zwischen Zahnarzt und Patient. *Johan Peter Woelber, Constanze Lessing und Dietmar Oesterreich *plädieren für eine sprechende Zahnmedizin als integraler Bestandteil der zahnmedizinischen Versorgung und zeigen Wege auf, wie dieses Konzept in Aus‑, Fort- und Weiterbildung sowie in der täglichen Praxis umgesetzt und durch Anreize gefestigt werden könnte. Auch hierzu ist weitere Forschung nötig, beispielsweise zu den populationsbezogenen Kosten im Verhältnis zum Nutzen.

Die Inanspruchnahme und Kosten zahnmedizinischer Versorgung nehmen *Michael H. Walter und Michael Rädel *in den Blick. Sie können hierzu auf die Abrechnungsdaten einer gesetzlichen Krankenkasse für die Jahre 2010 bis 2018 zurückgreifen und Inanspruchnahmeraten für verschiedene zahnärztliche Leistungen und die damit verbundenen Kosten ermitteln. Es dominiert eine interventionsorientierte zahnmedizinische Versorgung, auch wenn für präventive Maßnahmen (Früherkennungsuntersuchungen bei Kindern) im hier betrachteten Zeitraum ein Anstieg der Inanspruchnahme beobachtet werden konnte. Ausgehend vom zugrunde liegenden Vergütungssystem sind hier nach Ansicht der Autoren keine substanziellen Änderungen zu erwarten. Die Kosten sind insgesamt nur moderat gestiegen, allerdings sind die vom Patienten selbst getragenen Kosten hierbei nicht berücksichtigt.

Das Heft schließt mit einem Plädoyer von *Hans Jörg Staehle* für eine „frugale“ Zahnmedizin. Damit sind zahnärztliche Maßnahmen angesprochen, bei denen unter Berücksichtigung der Patientenbedürfnisse kostengünstigere Alternativen bei gleicher oder auch besserer Qualität bestehen, bzw. Maßnahmen mit besserer Qualität bei neutralen Kosten. Der Autor zeigt auf, welche Schritte in Forschung, Lehre und Patientenversorgung zielführend sein könnten, um frugale Interventionen nachhaltig zu etablieren. Dieses Konzept der frugalen Zahnmedizin scheint nicht zuletzt anschlussfähig an das Konzept der wertebasierten (Mund‑)Gesundheitsversorgung („value-based (oral) health care“; [1, 2]).

Liebe Leserin, lieber Leser, wir hoffen, dass wir mit den hier ausgewählten Themen Ihr Interesse an dem Thema Mundgesundheit wecken konnten, und wünschen Ihnen eine anregende Lektüre. Bleibt uns noch, uns bei den Autorinnen und Autoren sowie bei allen, die Ihre Zeit für die Begutachtung zur Verfügung gestellt haben, zu bedanken.
